# Different types of acupuncture and moxibustion therapy for neurogenic bladder after spinal cord injury: A systematic review and network meta-analysis study protocol

**DOI:** 10.1097/MD.0000000000018558

**Published:** 2020-01-03

**Authors:** Hanzhou Lei, Yanan Fu, Guixing Xu, Zihan Yin, Ling Zhao, Fanrong Liang

**Affiliations:** The Acupuncture and Tuina School, Chengdu University of Traditional Chinese Medicine, Chengdu, Sichuan, China.

**Keywords:** acupuncture and moxibustion therapy, network meta-analysis, neurogenic bladder dysfunction, protocol, system review

## Abstract

**Background:**

The invasive surgical intervention for neurogenic bladder dysfunction (NBD) following spinal cord injury (SCI) involves permanently altering one's body system and carries many surgical related risks and medication side effects are often seen in long term usage of pharmaceutical medications. Therefore, acupuncture and moxibustion therapies have been recommended due to their efficacy, simplicity of operation, cost effectiveness and safety. This protocol is designed for systematic review and network meta-analysis, which will perform comparisons or rankings of efficacy among the currently available acupuncture and moxibustion techniques and provide evidence to guide the best practice in acupuncture and moxibustion treatments of NBD due to SCI.

**Methods/Design:**

The Cochrane Library, EMBASE, PubMed, Web of Science, CENTRAL, CNKI, The VIP Database, The Wanfang database, CDFD, CMFD will be searched from inception to November 1, 2019. All randomized controlled trials containing eligible interventions(s) and outcome(s) will be included. The quality of included trials will be assessed using the “Risk of bias” tool from the Cochrane Handbook (V.5.1.0). Data analysis will be conducted by using STATA software (Version 13.0). Continuous outcome will be indicated as mean difference (MD) or standard mean difference (SMD), and enumeration data will be presented with odds risk (OR) or relative risk (RR).

**Results:**

This systematic review and network meta-analysis study aims to determine the most effective and safe approach in relieving urinary symptoms, and whether it produces better results in urodynamic examination. And a high-quality ranking of the therapeutic classes will be presented. The report will follow the PRISMA checklist for network meta-analysis. Results of the search strategy and the study selection will be presented in a PRISMA compliant flow chart.

**Conclusion:**

This study aims to propose a standard clinical decision-making guideline for acupuncture and moxibustion treatment of NBD after SCI.

## Abstract 2

Supplemental Digital Content is available in the text

## 1 Introduction

Spinal cord injury (SCI) affects more than 250,000 people in United States, with an estimate of 10000 to 12000 new diagnosis annually. Neurogenic bladder dysfunction due to SCI associated within continence, urinary tract infection, renal impairment, kidney stones, and poor quality of life, can significantly threat one's wellbeing.^[1]^ Today the mortality rate due to renal failure after SCI has dramatically declined, however, the management of NBD related symptoms and poor quality of life remain to be challenging for healthcare professionals.^[2]^ Disturbances of micturition are very common among SCI individual, more than 80% of the cohort experience at least some degree of bladder dysfunction.^[1]^ Baseon the locations of the spinal cord lesions, the neurogenic bladder dysfunctions can be classified as either upper motor neuron dysfunctions(injuries above S1) or lower motor neuron dysfunctions(injuries at S1-S4). The patho-mechanisms are multifaceted depending on the location and completeness of the injury. In upper neuron dysfunctions, the spinal cord injury can cause suprasacral neurogenic detrusor overactivity (NDO)associated with poorly sustained bladder contractions, discoordinated urethra and bladder, involvement of C-fiber in micturition reflex and overactive bladder and/or external sphincter sensitive to previous irrelevant stimuli such as penis, consequently resulting in high detrusor pressure, retention and incontinence. Meanwhile, the spinal lesion may also lead to detrusor-sphincter dyssynergia (DSD), in which bladder outlet obstruction occurs as the result of increased sphincter activity during detrusor contraction. This bladder outlet obstruction together with the high, sustained intravesical pressure caused by NDO is the major factors for renal damage and failure. For individuals with lower motor neuron dysfunction, the injury may cause parasympathetic decentralization of the bladder detrusor. The somatic nerves of the external urethral sphincter and some of afferent pathways may also be affected, leading to the loss of micturition reflex and sensation of bladder fullness.^[1]^

In western medicine treatment of NBD related to SCI, the goals are to reduce urinary complications, subsequently preserve renal functions and improve quality of life. Clean intermittent catheterization (CIC) is considered as the gold stand modality to manage urinary symptoms related to NBD.^[1]^ However, the outcomes maybe limited by poor compliance or the patients’ ability to cope with the manoeuvre. While the Suprapubic cystostomy(SPC), may be the ideal means for some of these patients, this long-term indwelling catheter also increase risk of bleeding, infection and accelerated renal deterioration. Surgical and pharmaceutical interventions are both well documented to be effective for NBD. However, the invasive surgical intervention involves permanently altering one's body system and carries many surgical related risks.^[5]^ And medication side effects are often seen in long term usage of pharmaceutical medications.

Acupuncture and moxibustion therapies are significant components of Traditional Chinese Medicine, which had been used for thousands of years. These traditional modalities are popular among the Chinese population due to their efficacy, simplicity of operation, cost effectiveness, and safety. There is also an increasing global interest and demand for these therapies in modern day society.^[6]^ In 1979, the World Health Organization (WHO) drafted out a provisional list of 47 diseases that could be treated by means of acupuncture therapy, including NBD following SCI.^[7,8]^

Preclinical studies have demonstrated that acupuncture can activate nerve regeneration, stimulate, and/or regulate of the nerve conduction in damaged nerves.^[9,10]^ Because NBD is a type of disturbances of micturition caused by neuropathy or damage, often accompanied by a coordinated disorder of bladder and urethral function, so acupuncture can alleviate the bladder/urethral dysfunction(maximum urinary flow rate (Qmax), post voiding residual urine volume (RUV) and maximal detrusor pressure, etc.) and trigger micturition through stimulating specific nerves. For instance, stimulation of SP6 can prolong the inter-contraction interval (ICI) in rats with overactive bladder^[11]^ and reduce c-Fos expression in certain specific areas of brain^[11]^ wherein c-Fos expression increases in response to bladder stimulation. Also, the synthesis and release of neurotransmitters may be affected by acupoint-stimulation, for instance substance P, nitric oxide synthase and calcitonin gene-related peptide.^[15–17]^ Clinical trials indicated that acupuncture therapy can increase maximum bladder capacity and bladder compliance. Likewise, electro-acupuncture can relieve overactive bladder symptoms, including the first sensation of bladder filling, first urge to void, and maximum cystometric capacity.^[18,19]^ In general, the management of neurogenic bladder after SCIis based on the basic principles of acupuncture and moxibustion therapies, in which stimulating acupuncture points to regulate the physiological function of the bladder.

Many investigations have been conducted before to evaluate the efficacy of available symptom-relief interventions. While most of previous systematic reviews and meta-analyses were focusing on the safety and efficacy of acupuncture in relieving urinary symptoms^[20,21]^ there are lack of comparisons or rankings of efficacy among the currently available acupuncture and moxibustion therapies for neurogenic bladder due to SCI.

This protocol is designed for systematic review and network meta-analysis, which will overcome the constraints by creating indirect contrasts and implementing data composing^[22,23]^ provide evidence to guide the best practice in acupuncture and moxibustion treatments of neurogenic bladder due to SCI.

The present systematic review and network meta-analysis aims to:

1. Propose a ranking of the currently available acupuncture and moxibustion therapies in patients with neurogenic bladder following a spinal cord injury. The comparisons of safety and efficacy among different approaches from in the obtainable clinical studies will be performed by using pair-wise meta-analysis and a Bayesian random model network meta-analysis;
2. Investigate the relevance between spinal cord injury level and curative effect. When possible, both the short-term and the long-term effects will be evaluated according to the urodynamic examination measurement indexes of urination diary.
3. Determine/demonstrate the most effective and safe approach in relieving urinary symptoms, and whether it produces better results in urodynamic examination.

## 2 Methods

**Figure 1:**
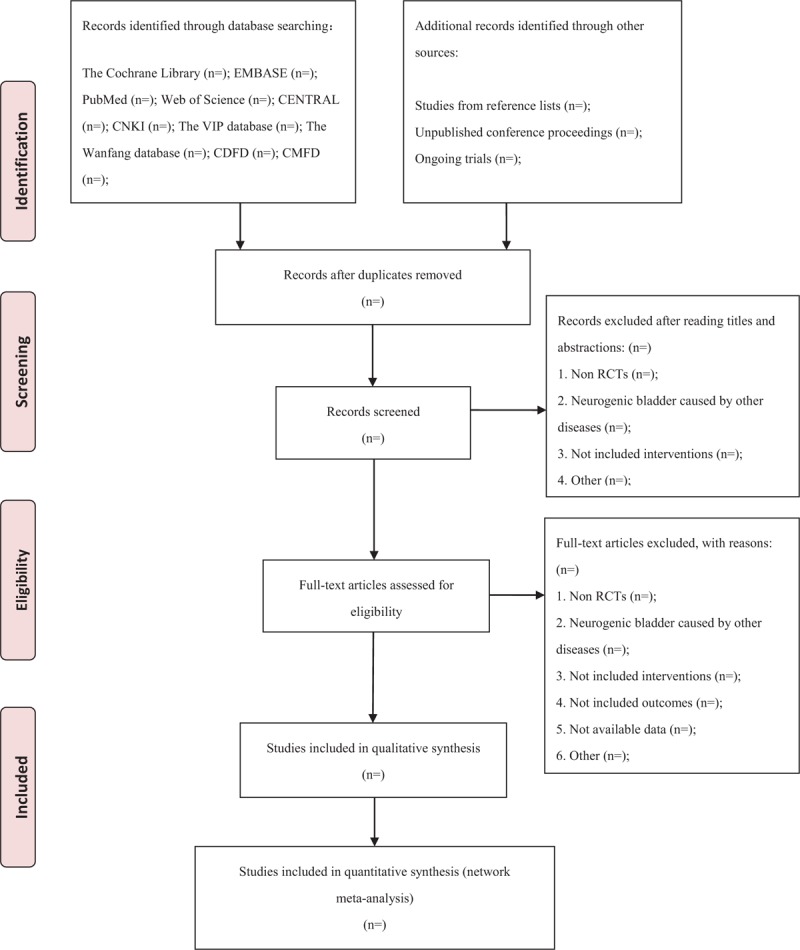
PRISMA flow chart. (The trial selection process of the proposed protocol, according to the guidelines of PRISMA-NMA.).

This protocol has been registered on the international prospective register of systematic review (PROSPERO), and the trail registration number is CRD42019128508. This systematic review and network meta-analysis will be designed according to the guidelines of preferred reporting items for systematic reviews and network meta-analysis (PRISMA-NMA).^[24]^ The trial selection process is shown in PRISMA flow diagram (Fig. 1).

### 2.1 Criteria for considering studies for this review

#### 2.1.1 Types of studies

All randomized controlled trials (RCTs) containing eligible interventions(s) and outcome(s) will be included. The index tests of studies must include either acupuncture, electro-acupuncture, TENS, fire needle, body acupuncture, warm needle, auricular acupuncture, scalp acupuncture, elongated needle, intradermal needle and moxibustion, or combinations thereof;

Conference proceedings and abstracts might be included if feasible. In order to exam the duplicate publications of the same study and obtain the most relevant and latest paper with complete data, each available paper will be retrieved by reference list.

Studies will be excluded if they consist with the following elements: reviews, commentaries, short surveys, case reports, and letters retrospective or prospective non-randomized studies, self-prospective studies, non-randomized cross-over studies and purely studies on the mechanism of acupuncture and moxibustion, regardless of publication status.

The language limitation of the publications included is Chinese and English.

#### 2.1.2 Types of participants

Patients of any age and gender with a clear diagnosis of neurogenic bladder disease resulted from spinal cord injury (timing or cause immaterial), and could be on acupuncture and moxibustion treatment. The diagnosis of disease should have been established using standard criteria or, if necessary, by the definition of neurogenic bladder following SCI given by the author in the corresponding clinical trial.

Studies will be excluded if they contained following elements:

1. Study objects with neurogenic bladder disease that are caused by conditions such as diabetes, stroke, craniocerebral injury, cancers, central nervous surgery or extensive pelvic surgery, encephalitis, mymelitis, drug effects, and congenital diseases.
2. Participants who had previous surgeries for neurogenic bladder.

#### 2.1.3 Types of interventions

The Interventions of treatment group will include all types of acupuncture and moxibustion approaches, for instance, acupuncture, electro-acupuncture, fire needle, body acupuncture, warm needle, auricular acupuncture, scalp acupuncture, elongated needle, intradermal needle, and moxibustion, etc. Acupuncture combined with other conservative treatments will also be included (combined interventions consisting of 4 or more therapies or with potential safety problems will be excluded.),without restrictions of acupuncture and moxibustion manipulation methods, acupoint selection, materials of interventions, needle retaining time, course of treatment, and follow-up period.

The control group will include treatments of non-acupuncture and non-moxibustion method, such as sham acupuncture, medication/drugs, rehabilitation(for example, bladder training, pelvic-floor muscle exercises, pelvic-floor electro-stimulation and biofeedback), external appliances(condom catheters, pads or penile clamps), and other conservative treatments such as catheterisation (intermittent or indwelling), etc.

#### 2.1.4 Types of outcome measures

##### 2.1.4.1 Primary outcomes

The primary outcome is the difference in urinary symptoms before and after treatment, as reported by participants in a voiding diary or a self-report questionnaire. It includes the mean number of urination and/or incontinence episodes per 24 hours, the number of participants with incontinence or retention, and the number of participants requiring catheterisation.

##### 2.1.4.2 Secondary outcomes

The secondary outcomes include the following items:

1. changes in urodynamic tests before and after treatment, for example, maximum urinary flow rate (Qmax), postvoiding residual urine volume (RUV), and maximal detrusor pressure;
2. change in clinical assessment before and after treatment, for example, the standardised pad test (1 hour or 24 hours; quantified leakage), severity of incontinence, and incidence of recurrent urinaryin continence or retention;
3. change in a QoL questionnaire before and after treatment, for example, condition-specific QoL measures, general health QoL measures and psychologically related scales; and
4. the effective rate. Safety outcome measures include the incidence of all reported adverse events such as local pain, haematomas, fainting during acupuncture treatment, and complications related to neurogenic bladder.

### 2.2 Search methods for identification of studies

#### 2.2.1 Electronic searches

The following database resources will be used for the identification of trials:

- The Cochrane Library
- EMBASE
- PubMed
- Web of Science
- The Cochrane Central Register of Controlled Trials (CENTRAL)
- The China National Knowledge Infrastructure (CNKI)
- The VIP Database for Chinese Technical Periodecals
- The Wanfang database
- The China Doctoral Dissertations Full-text Database (CDFD)
- The China Master's Theses Full-text Database (CMFD)

The last search for all databases was updated to November 1, 2019, and studies published in Chinese and English publications will be included.

The detailed search strategy of PubMed will be shown in Table 1.

**Table 1:**
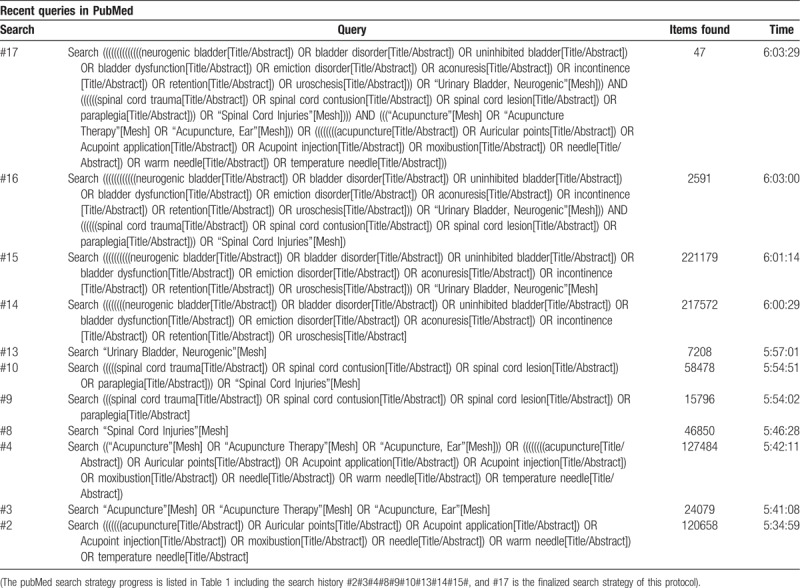
Detailed search strategy in pubMed databases.

#### 2.2.2 Searching other resources

In addition, studies acquired from the reference lists of all eligible studies, relevant reports of clinical trials and review articles will also be included. We will also be searching for the eligible ongoing trials through the WHO international clinical trials registry platform (http://apps.who.int/trialsearch/), the National Research Register (http://www.update-software.com/projects/nrr/) and ‘Current Controlled Trials’ (http://www.controlled-trials.com with links to other databases of ongoing trials).

### 2.3 Data collection and analysis

#### 2.3.1 Selection of studies

The search strategy will be formulated by 3 investigators (LHZ, FYN, and XGX) and the search results will be store by 1 author (ZL) in Endnote X9.ALLpreviousdatabaseretrievals will then be checked by the 2 reviewers (XGX and YZH).

The 2 reviewers (XGX and YZH) will independently identify and select the searched clinical studies according to the inclusion criteria. An abstract screening process will be performed to remove duplicate records and unqualified articles, and the full-text articles will be assessed for eligibility. The citation lists of eligible articles will also be cross checked for potential eligible studies. For each excluded study, reason (s) for exclusion will be given.

In cases of conflicting opinions, face-to-face discussions will be conducted with other reviewers to reach for general consensus.

#### 2.3.2 Data extraction and management

Two independent investigators (XGX and YZH) will extract data from the studies that are eligible for full-text assessment. If any discrepancy appears, a third reviewer (LFR) will also examine the data. The multi-arm trials will be split into dual-arm trials in which the results can be composed.^[25]^

Eligible extracted items

1. General information (author, working location, publication date, publication source, etc.);
2. Characteristic of trial (design of the study, number of groups, number of participants for treatment and control, method of randomization, blinding, method of analysis, objectives of the study, etc.)
3. The participants (age, gender, ethnicity, country, diagnosis, duration, etc.);
4. characteristics of interventions and controls (method of the intervention, number of treatment, frequency of treatment, duration of a session, name and type for control, information of caring, additional treatment, etc.);
5. Outcome measurements (primary outcome and secondary outcome according to types of outcome measures, timeline for assessment, length of follow-up, etc.);
6. Results (mean, SD, observed events after intervention, total sample size, etc.);
7. Risk of bias (randomization, allocation concealment, incomplete data, blinding, selective report, conflicts of interest, and other bias).

All extracted data will be saved in an Excel spreadsheet developed in light of recommendations in the Cochrane Handbook.

#### 2.3.3 Dealing with missing data

Reviewers will contact the corresponding authors of the studies by telephone, email or post to collect the missing data, if the required data are ambiguous or not reported in the included articles. And the missing data will be collected by using the data extraction form.

#### 2.3.4 Assessment of risk of bias in included studies

The risk of bias will be assessed using the “Risk of bias” tool from the Cochrane Handbook (V.5.1.0),^[26]^ the risk of bias of the RCTs will be categorized into “high risk”, “low risk” and “unclear risk”.

The “Risk of bias” template will be attached as the supplementary materials of analysis results which consist the following 6 major items:

1. selection bias: random sequence generation and allocation concealment;
2. performance bias: blinding of investigators, participants, and care providers;
3. detection bias: blinding of outcome assessment;
4. attrition bias: incomplete data/differential dropout;
5. reporting bias: selective reporting;
6. other bias: for example, conflict of interest, follow-up, non-intention-to-treat or per-protocol analysis, etc.

#### 2.3.5 Assessment of heterogeneity

The clinical, statistical, and methodological differences may be attributed to heterogeneity. Statistical heterogeneity will be analyzed by the Mantel-Haenszel χ^2^ test for heterogeneity. A calculation with a *P* value <.10 and a high *I*^2^ value indicates statistically significant heterogeneity. On the basis of the Cochrane Handbook,^[26]^*I*^2^ values can be classified into 4 categories: a value of 0% to 40% indicates little or no heterogeneity; a value of 30% to 60% indicates moderate heterogeneity; a value of 50% to 90% indicates substantial heterogeneity; and a value of 75% to 100% indicates considerable heterogeneity. Statistical heterogeneity also depends on the magnitude of effects, the direction of results and the strength of evidence.

The pair-wise meta-analysis will be performed to synthesize studies that compared the same interventions with random effects models (direct comparison) using the STATA software (Version 13.0).

#### 2.3.6 Assessment of Inconsistency

For the consistency among the included trials is a basic principle used to conduct network meta-analyses, this result generated by an indirect comparison should be similar compared to the result derived from a direct comparison. The Z test will be performed to analyze the inconsistencies. A Z-value and its corresponding *P*-value will be calculated, and if *P* value less than .05 indicates statistical significant difference, the inconsistencies exist in the indirect comparison.^[27]^

#### 2.3.7 Planned methods of analysis

Bayesian random model network meta-analysis:

A random effects model network meta-analysis (combination of direct and indirect comparison) will be developed in a Bayesian frame work using a Markov Chain Monte Carlo method provided by the STATA software.^[28,29]^ The prior distribution will be chosen as non-informative. The posterior distribution will be estimated using a Markov Chain Monte Carlo method.

The direct and indirect comparisons for each pair of treatments will be merged by modeling the continuous outcomes in every treatment group of included studies. And, the Brooks-Gelman-Rubin method will be included to assess the convergence between direct and indirect variances in the relative process.^[30]^

Subgroup analysis:

We will be performed subgroup analysis to search the potential results of inconsistence and heterogeneity, such as the levels of SCI etc.

#### 2.3.8 Geometry of the network

Network plots will be obtained by STATA software. The results of the effectiveness of different types of acupuncture and moxibustion treatments will be demonstrated by: the size of the nodes in network plots is proportional to the number of studies evaluating intervention; the thickness of the lines between the nodes is proportional to the number of direct comparisons between interventions.

The network is based on a netlike association analysis because there exists at least 1 study evaluating an available therapy together with at least 1 of the other remaining treatments.^[31]^ A loop connecting 3 interventions shows that there is at least 1 study comparing the 3 targeted interventions simultaneously.

#### 2.3.9 Summary measures

For dichotomous data, the results of each study will be pooled and presented as a risk ratio with 95% CIs. A mean difference with 95% CI will be used for continuous data.^[26]^

According to the network plot and network estimates graph, the proportion contribution of direct comparisons will be unfolded. Also, different direct comparisons in network meta-analysis estimates have different effects on the result, so we can assess the extent to direct comparisons of different control measures impact the combined results of network meta-analysis by drawing network meta-analysis estimates graph.

In addition, the forest plot will demonstrate the combined network meta-analysis results, including the treatment effects of different techniques by mean with 95% CI and 95% Prl and the prediction interval. Then, the effectiveness of different techniques will be ranked by plots of the cumulative ranking curve (SUCRA) by means of the STATA software.^[32]^ The results will be expressed in percentage terms, and the closer the value is to 100%, the better the curative effect.

#### 2.3.10 Report bias across studies

The Deek funnel plot will be applied to evaluate the potential publication bias where there are more than 10 studies available for an index test.^[33]^

## 3 Results

The report will follow the PRISMA checklist for network meta-analysis.^[24]^ Results of the search strategy and the study selection will be presented in a PRISMA compliant flow chart.^[34]^ Study characteristics will be summarized, as shown in Table 2 (Supplemental Digital Content). Treatment effects will be summarized with OR estimates and their 95% credible intervals. A ranking of the therapeutic classes will be presented.

## 4 Conclusion

The network meta-analysis is capable of synthesizing direct and indirective evidence and analyzing the interrelations of multiple interventions. Therefore, the results of the analysis can determine the best therapeutic regimen for clinicians and patients, and can visually indicate the comparison results of effectiveness and safety among the available techniques. This study will be proceeded in accordance with this protocol, strictly controlling the quality of the included studies and the similarity of the basic characteristics of the study. Our review will contribute to public health and clinical research for further investigations of complementary and alternative medicine for neurogenic bladder after spinal cord injury.

## Acknowledgments

The authors thank Mr. Ze-peng Wei (Haematology Ward Royal North Shore Hospital Australia, helped us to revise the English of the manuscript.

## Author contributions

XXX

## Supplementary Material

Supplemental Digital Content
