# Supplementary material for: Different types of acupuncture and moxibustion therapy for neurogenic bladder after spinal cord injury: A systematic review and network meta-analysis study protocol
Source: Medicine (Baltimore). 2020 Jan 3;99(1):e18558. doi: 10.1097/MD.0000000000018558 (PMC6946264; doi:10.1097/MD.0000000000018558)
Supplement: Supplemental Digital Content [file medi-99-e18558-s001.docx]

(Supplemental Digital Content)**Table2. Characteristics of the included studies.**( The characteristics of the eligible trails will be extracted with following elements: PMID, author, title, publication, sample size, age, levels of spinal cord injury plane, baseline, diagnostic criteria, intervention, outcomes, duration, dropout cases, adverse event.)

| **PMID** | **Year** | **1st author** | **Title** | | **Publication** | | **Sample size**  **(T/C)** | **Age** | **Disease course** | **Levels of spinal cord injury plane (n)** | **Baseline** | **Diagnostic criteria** | **Intervention**  **(T)** | **(C)** | **Outcomes** | **Duration** | **Dropout cases** | **Adverse event** |
| --- | --- | --- | --- | --- | --- | --- | --- | --- | --- | --- | --- | --- | --- | --- | --- | --- | --- | --- |
|  |  |  |  |  | |  | |  |  |  |  |  |  |  |  |  |  |  |
|  |  |  |  | |  |  | |  |  |  |  |  |  |  |  |  |  |  |
|  |  |  |  | |  |  | |  |  |  |  |  |  |  |  |  |  |  |
|  |  |  |  | |  |  | |  |  |  |  |  |  |  |  |  |  |  |
|  |  |  |  | |  |  | |  |  |  |  |  |  |  |  |  |  |  |
|  |  |  |  | |  |  | |  |  |  |  |  |  |  |  |  |  |  |
|  |  |  |  | |  |  | |  |  |  |  |  |  |  |  |  |  |  |
|  |  |  |  | |  |  | |  |  |  |  |  |  |  |  |  |  |  |
|  |  |  |  | |  |  | |  |  |  |  |  |  |  |  |  |  |  |
|  |  |  |  | |  |  | |  |  |  |  |  |  |  |  |  |  |  |
